# Supplementary material for: Creation of energetic biothermite inks using ferritin liquid protein
Source: Nat Commun. 2017 Apr 27;8:15156. doi: 10.1038/ncomms15156 (PMC5414172; doi:10.1038/ncomms15156)
Supplement: Supplementary Information — Supplementary Figures. [file ncomms15156-s1.pdf]

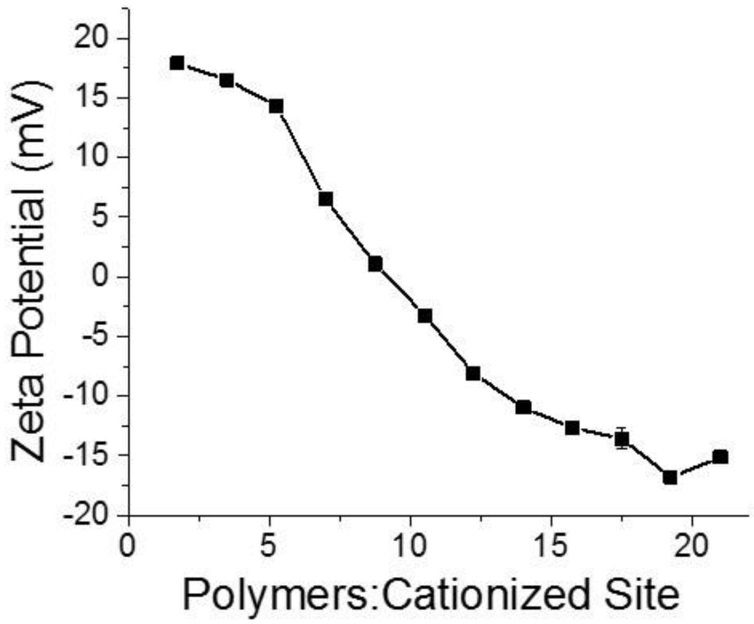

Supplemnetary Figure 1 │ Titration of cationized ferritin with polymer anion. Zeta potential plot of cationized ferritin with polymer anion in water.

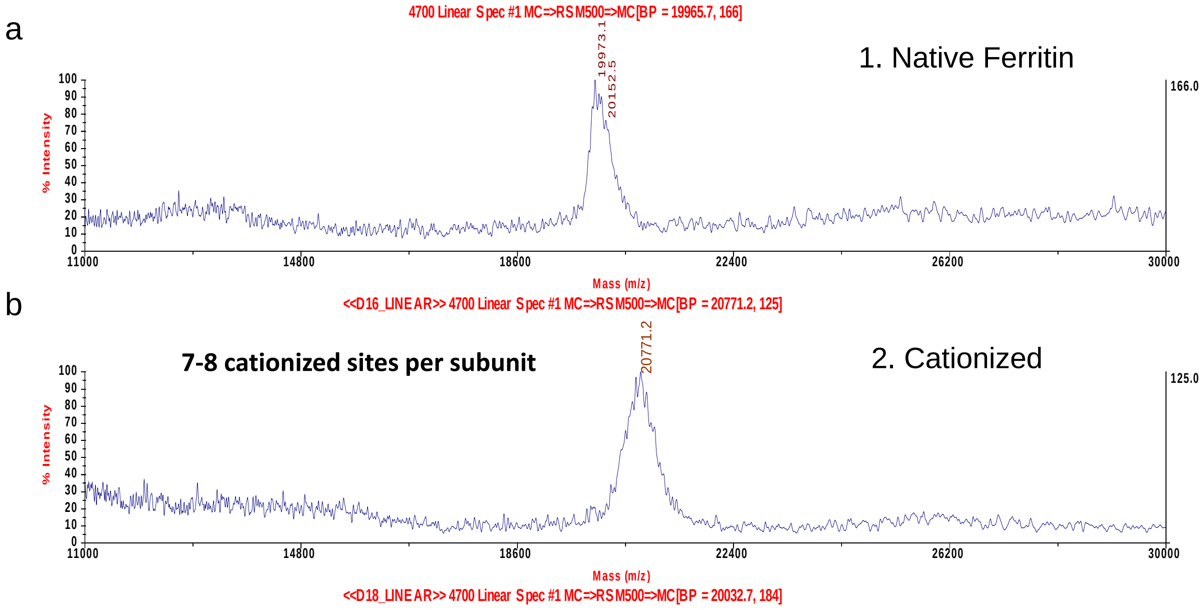

Supplementary Figure 2 | **Cationization of native ferritin.** **a**, MALDI mass spectrometry of Native ferritin heavy chain. **b**, cationized ferritin heavy chain. The MW of dimethylaminopropylamine (cationizing agent) is 102.18 g/mol.

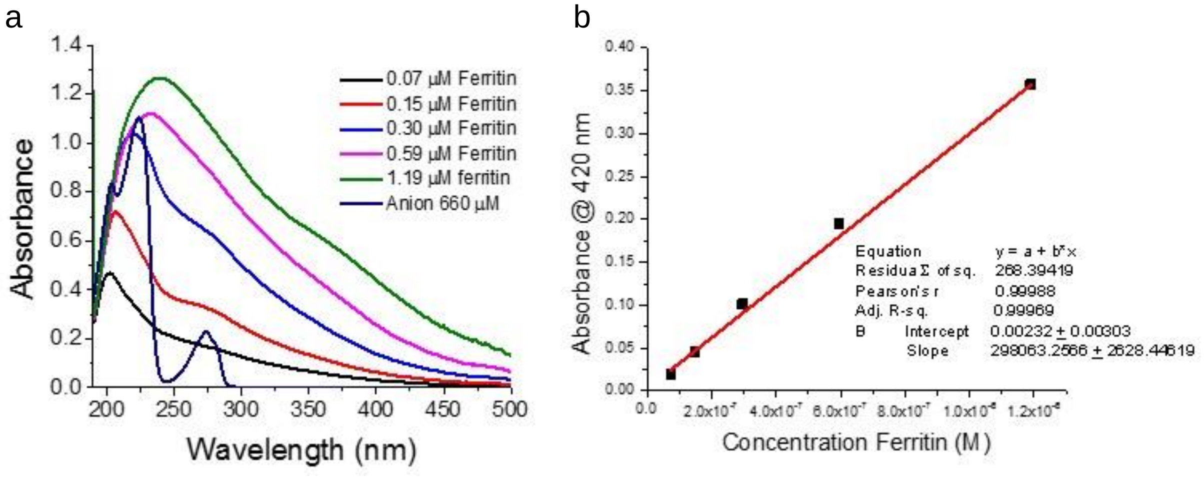

Supplementary Figure 3 | Calibration of ferritin concentration. **a**, Absorbance spectra of ferritin at different concentrations in water and polymer anion, separately. Polymer anion has no absorbance above 300 nm, while the iron oxide core of ferritin strongly absorbs at 420 nm. **b**, Standard concentration plot of ferritin vs absorbance. Consequently, to find the unknown concentration of ferritin in ferritin ionic liquid, we measured the absorbance at 420 nm of reconstituted ferritin ionic liquid in water in order to determine ferritin concentration and subtracted this converted amount from the initial total mass of solvent-free ferritin ionic liquid (no water) to find mass of polymer anion in final ferritin protein ionic liquid after dialysis. Finally, we calculated a ferritin:polymer anion stoichiometry based on these amounts. Absorbance spectra were collected on a Jasco J-815 CD spectrometer.

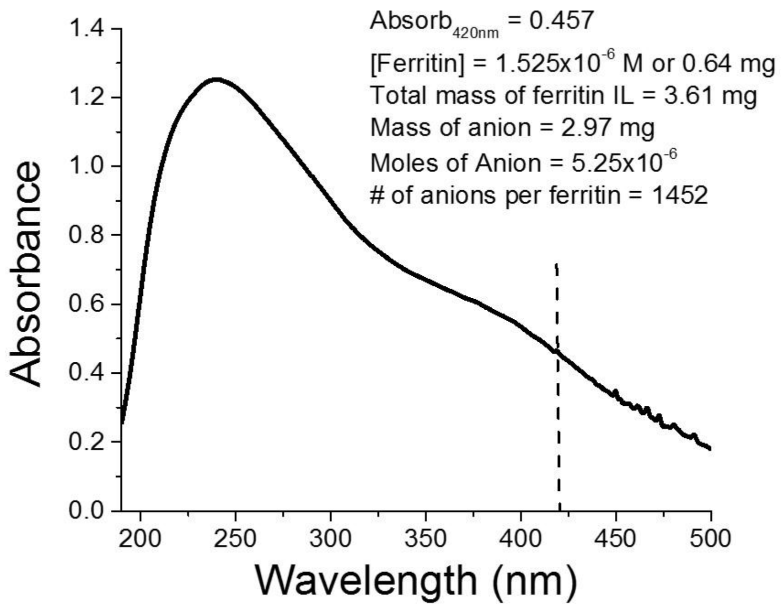

Supplementary Figure 4 | Quantitation of ferritin and anion components in ferritin ionic liquid. Absorbance spectrum of purified and dialyzed final ferritin ionic liquid reconstituted in water. 3.61 mg of dialyzed ferritin ionic liquid was reconstituted in 0.5 mL water and measured for absorbance. We determined the concentration of ferritin in the ferritin ionic liquid using absorbance at 420 nm and standard concentration plot in Supplementary Figure 3. The concentration of ferritin was converted into a mass and subtracted from the initial mass of the ferritin ionic liquid to yield the mass of polymer anion present in the ferritin ionic liquid.

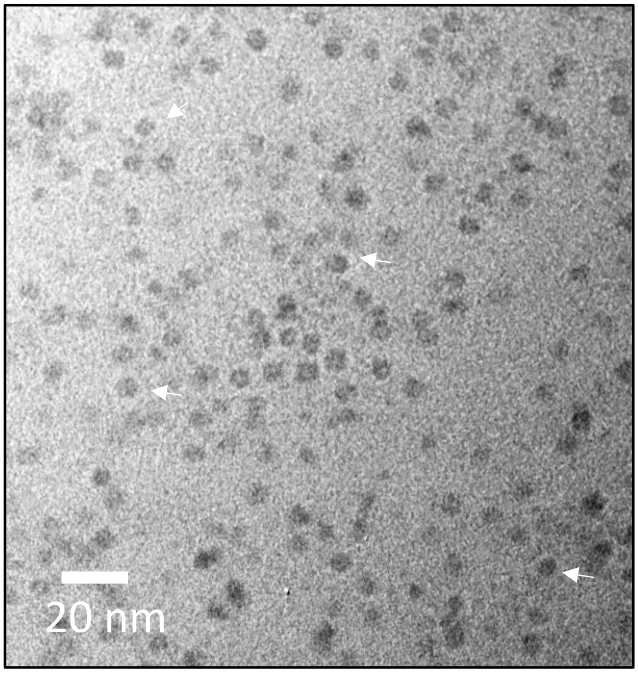

Supplementary Figure 5 │Stability of ferritin protein cage to sonication. TEM of reconstituted ferritin in water after sonication for 1 hour in a bath sonicator. Ferritin was negatively stained using UranylLESS stain for 10 minutes and imaged on a Phillips CM200 transmission electron microscope.

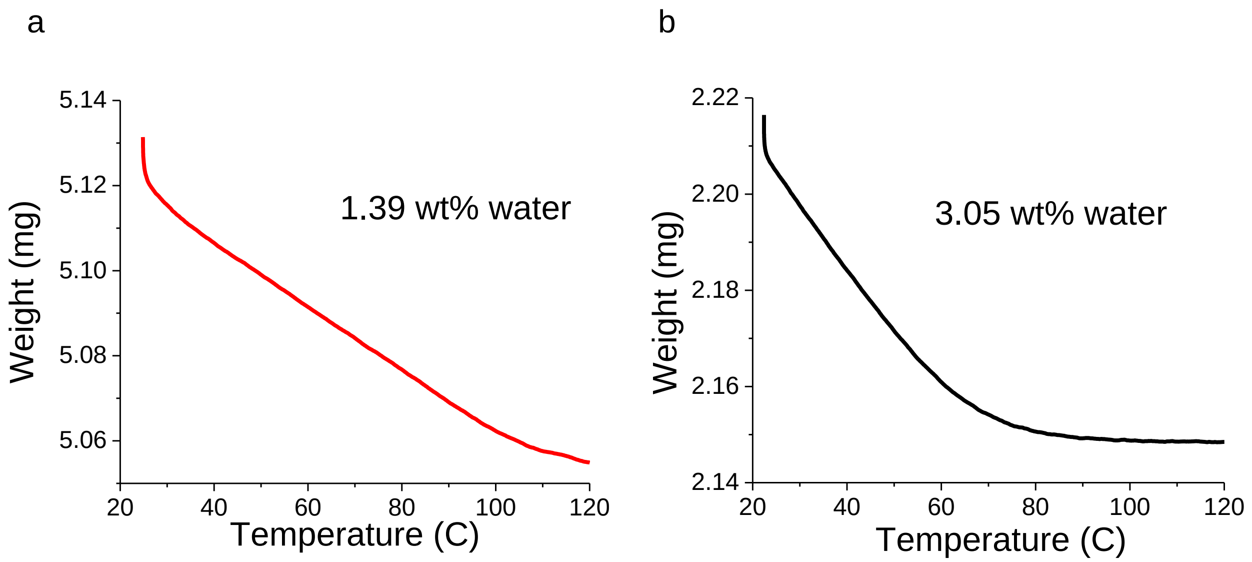

Supplementary Figure 6 │Determination of water content. **a**, Thermogravimetric analysis (TGA) plot of ferritin ionic liquid. **b**, TGA of nAl loaded ferritin ionic liquid showing water content.

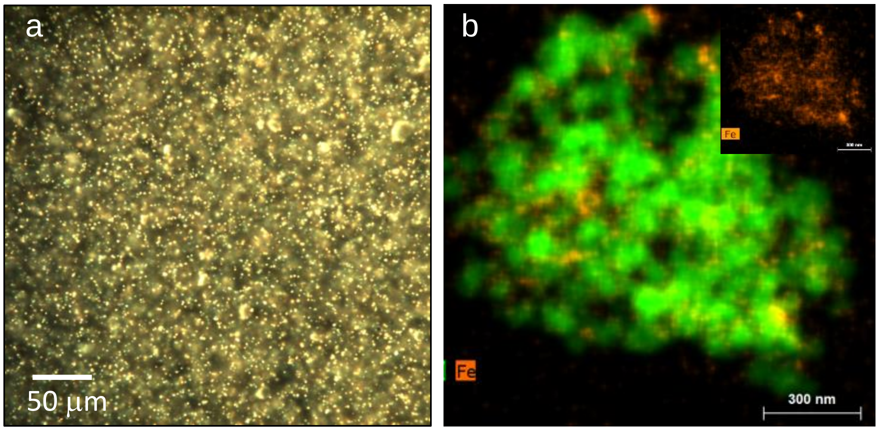

Supplementary Figure 7 │Characterization of nAl loaded ferritin ionic liquid. a, Dark field microscopy image of neat nAl@ferritin ionic liquid (no water). **b**, EDAX elemental map of nAl@ferritin ionic liquid after being reconstituted in water showing the presence of Al (green) and Fe (orange). Inset shows only iron (orange) of ferritin.

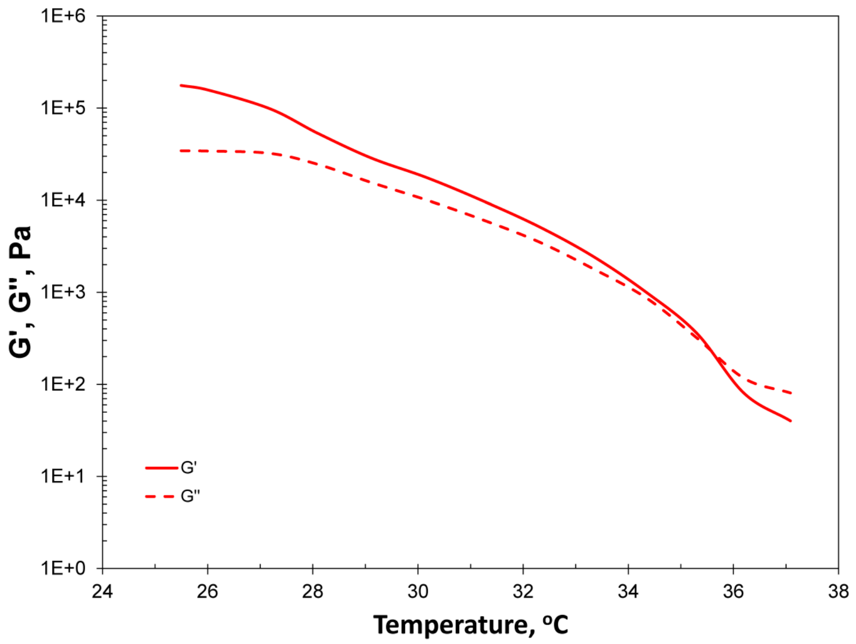

Supplementary Figure 8 │Rheology of nAl loaded ferritin ionic liquid. Thermal dependence of rheological properties of nAl dispersed in ferritin ionic liquid.

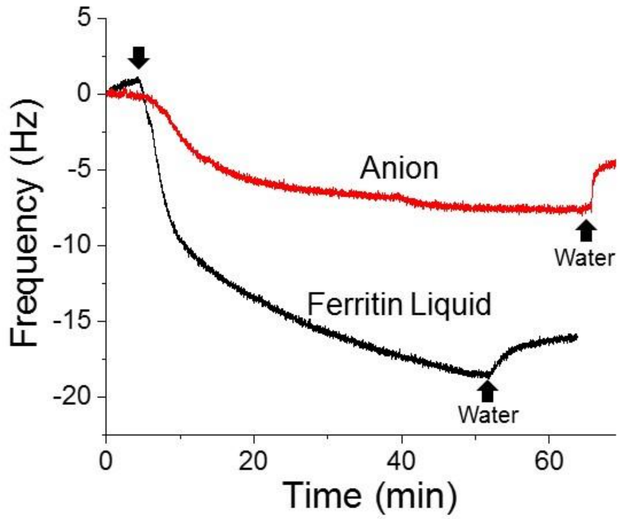

Supplementary Figure 9 │Binding of ferritin ionic liquid or anion to an alumina surface. Quartz crystal microbalance (QCM) plot of ferritin ionic liquid or anion only binding to an aluminum oxide coated QCM sensor (Q-Sense, QSX-309). A Q-Sense E4 QCM-D system with flow modules was used to measure binding. Flow rate was set at 0.17 mL/min and 3rd overtone frequency was measured.

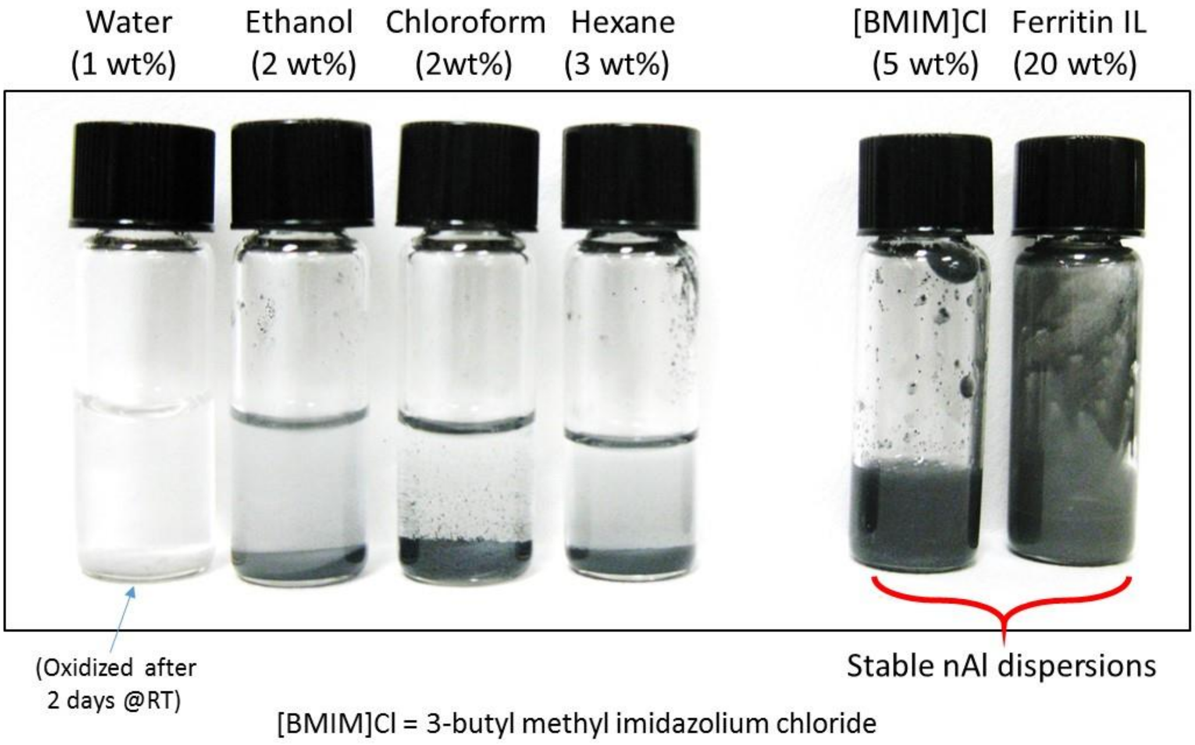

Supplementary Figure 10 | Dipersion of nAl in various aqueous, organic, and ionic liquid solvents. [BMIM]Cl = 3-butyl-1-methyl imidazolium chloride (Ionic Liquids Technologies). Water was doubly deionized while all other solvents were obtained from Sigma Aldrich. Briefly, 10 – 30 mg of nAl was added to solvents, sonicated for 10 minutes to produce a dispersion, and then left undisturbed on the benchtop for 2 days.

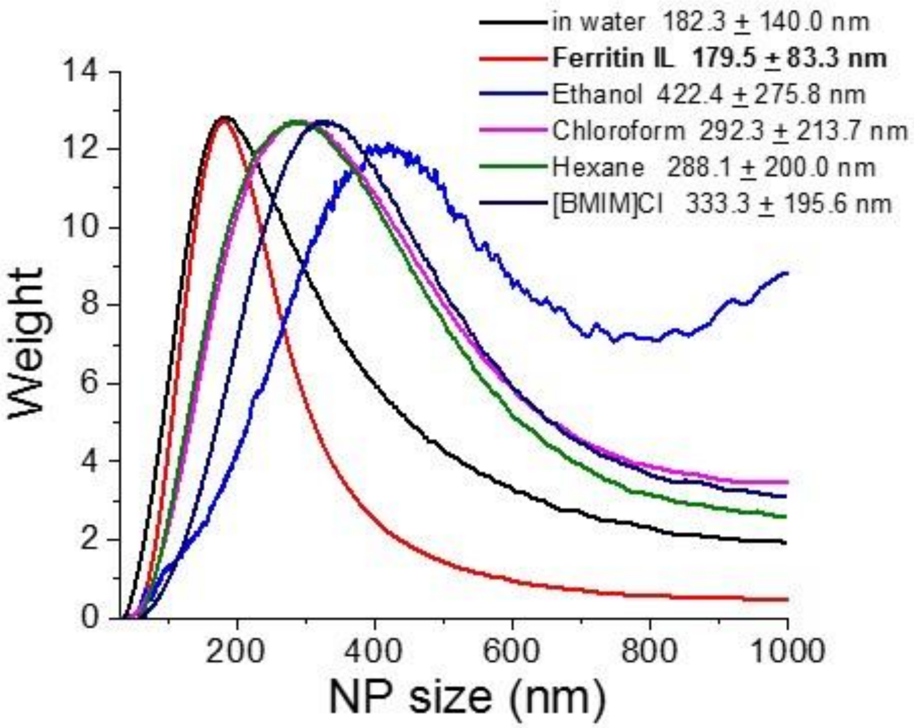

Supplementary Figure 11 | Particle size analysis of nAl dispersions. nAl size plots obtained using a CPS disc centrifuge particle size analyzer from CPS Instruments model DC24000UHR. Particle dispersions (100 µL of sample) were injected onto a sucrose gradient prepared using an auto sucrose gradient maker and 8% and 24% sucrose solutions. Sizes were automatically calilbrated during each run using an internal standard of 476 nm PVC spheres (CPS Instruments).

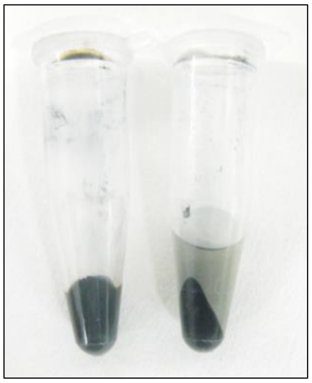

(Centrifuged @ 16873 x g for 1hr)

Supplementary Figure 12 | Dispersion stability of nAl in BmimCl and ferritin ionic liquid after centrifugation. nAl dispersions in BmimCl and ferritin ionic liquid were centrifuged for 1 hour at 16,873 x g.

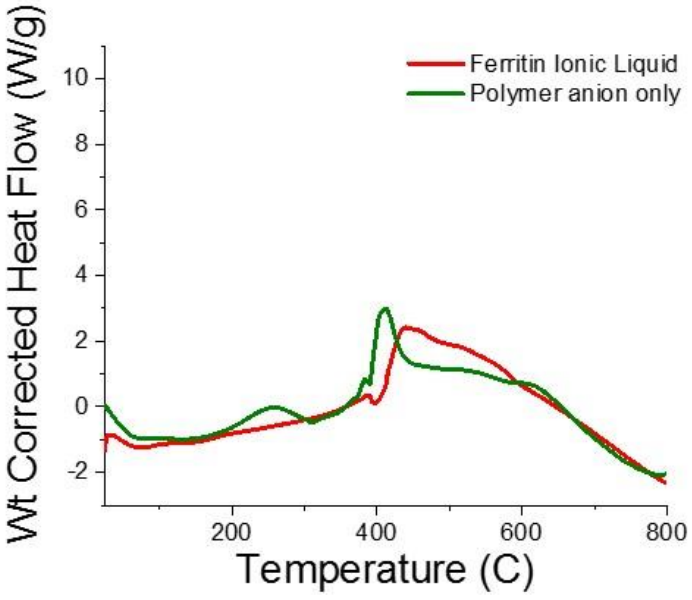

Supplementary Figure 13 | Heat profile of ferritin ionic liquid or polymer anion. Differential thermal analysis profile of ferritin ionic liquid and polymer anion under an Argon atmosphere and heating rate of 20°C/min. Heat of combustion for polymer anion was determined to be +2.8 kJ/g and -1.0 kJ/g for ferritin ionic liquid.

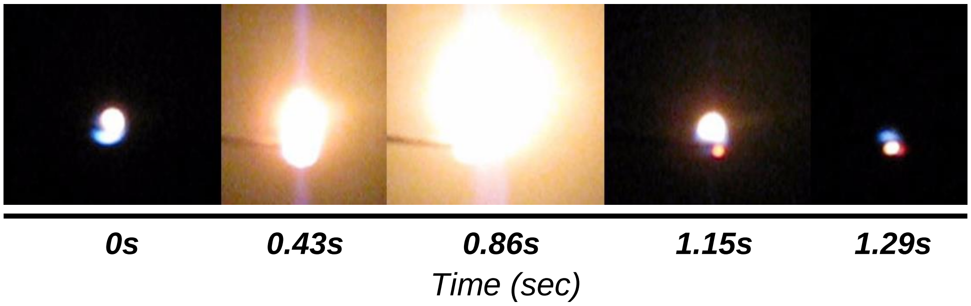

Supplementary Figure 14 | Combustion of nAl dispersed in an apoferritin ionic liquid (no iron oxide core). Combustion images of nAl@apoferritin ionic liquid (no iron oxide) ignited on a syringe tip.

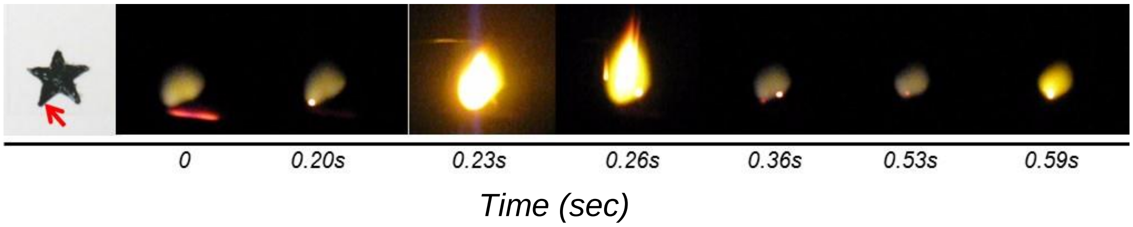

Supplementary Figure 15 | Combustion of patterned nAl@ferritin ionic liquid on glass. Combustion image of patterned nAl@ferritin ionic liquid hand drawn on a glass slide and ignited.

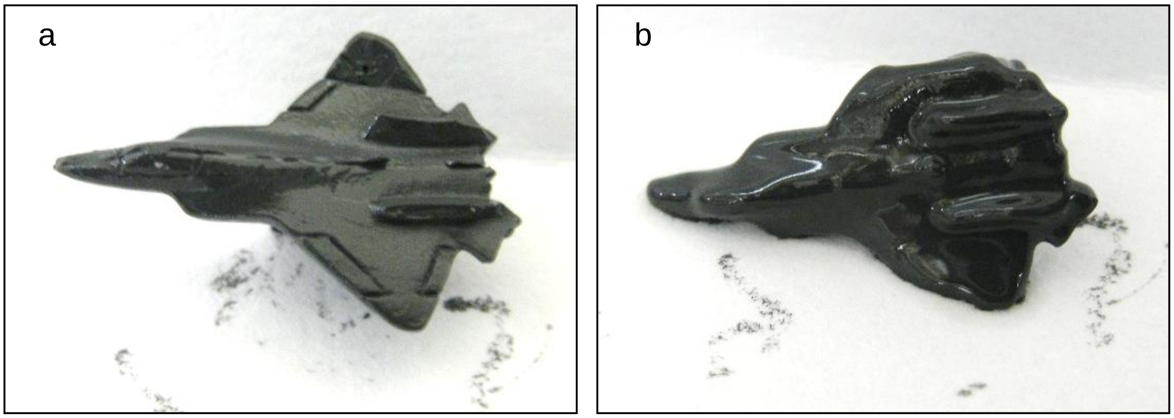

Supplementary Figure 16 | Phase stability of molded nAl@ferritin liquid after ageing. a, nAl@ferritin ionic liquid molded into an F-22 aircraft using a PDMS mold. **b**, Structure after 4 hours at room temperature exposed to air.

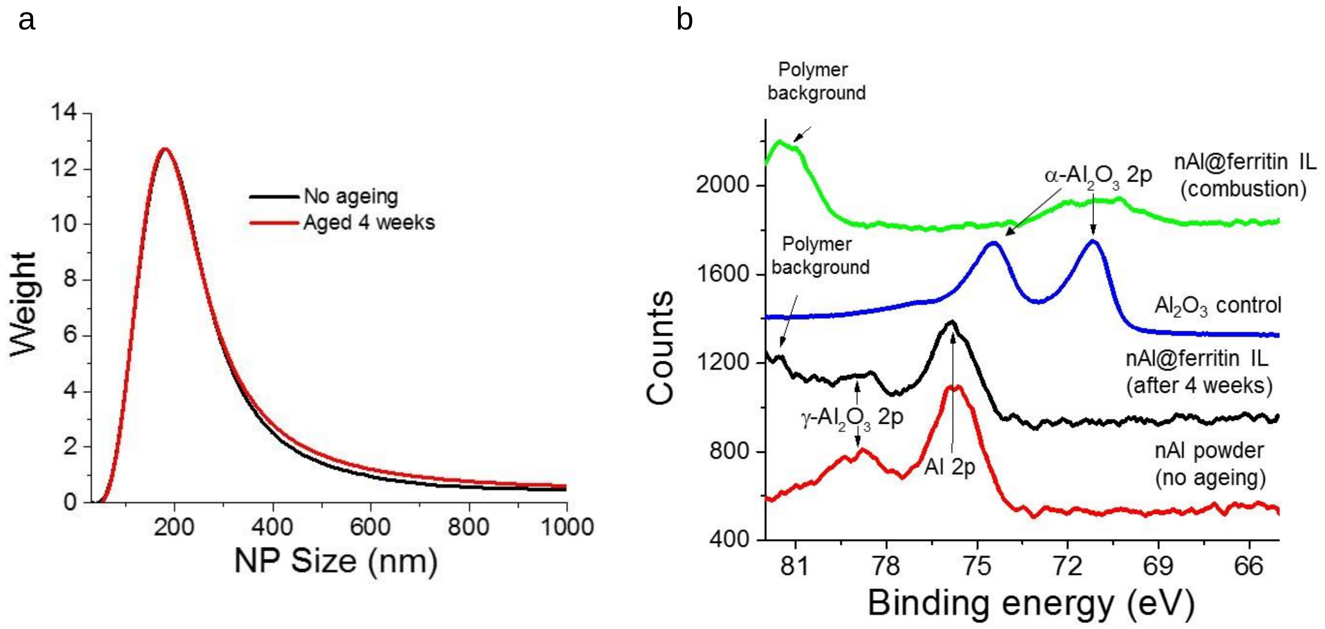

Supplementary Figure 17 | Oxidation and dispersion stability of nAl@ferritin ionic liquid after ageing. **a**, CPS particle size analysis of nAl@ferritin ionic liquid at day 0 and after 4 weeks. Samples were reconstituted in water and injected onto sucrose gradient. **b**, High resolution XPS spectra of nAl@ferritin ionic liquid after 4 weeks of ageing, nAl powder only (Day 0, no ageing), an Al_2O_3 sputtered surface (Biolin Scientific), and nAl@ferritin ionic liquid after combustion. XPS spectra were collected on an M-Probe Surface Science XPS spectrometer. Samples were prepared by dropcasting 1 µL of nAl@ferritin ionic liquid aged over 4 weeks, 1 µL of nAl dispersion in ethanol, or 1 µL of nAl@ferritin ionic liquid after combustion onto a silicon wafer and air dried. Spectra were collected using 0.01 eV steps and averaged over 100 scans.
